# Dr. Adelbert Dalton Atwood

**Published:** 1918-08

**Authors:** 


**Dr. Adelbert Dalton Atwood,** Buffalo 1879, died in Brooklyn, April 28, aged 68.

